# Retraction: Substance P and beta-endorphin mediate electro-acupuncture induced analgesia in mouse cancer pain model

**DOI:** 10.1186/1756-9966-28-137

**Published:** 2009-10-09

**Authors:** Hyo-Jeong Lee, Jae-Ho Lee, Eun-Ok Lee, Hyo-Jung Lee, Kwan-Hyun Kim, Sun-Hyung Kim, Keun-Sung Lee, Hee-Jae Jung, Sung-Hoon Kim

**Affiliations:** 1 College of Oriental Medicine, Kyung-Hee University, Seoul 130-701, South Korea; 2 Medical center, Kyung-Hee University, Seoul 130-701, South Korea

## Retraction

Following the publication of this article [1] in *Journal of Experimental and Clinical Cancer Research*, the corresponding author has informed the journal that this article had been accepted and previously published by *Acupuncture and Electro-therapies Research *[2]. Since it has been brought to the attention of all authors the decision has been made to retract the article published in *Journal of Experimental and Clinical Cancer Research*. The authors apologise for any inconvenience this may have caused to the editorial staff and readers.
